# Mapping local hot spots with routine tuberculosis data: A pragmatic approach to identify spatial variability

**DOI:** 10.1371/journal.pone.0265826

**Published:** 2022-03-24

**Authors:** Meredith B. Brooks, Ana Karina Millones, Daniela Puma, Carmen Contreras, Judith Jimenez, Christine Tzelios, Helen E. Jenkins, Courtney M. Yuen, Salmaan Keshavjee, Leonid Lecca, Mercedes C. Becerra

**Affiliations:** 1 Department of Global Health and Social Medicine, Harvard Medical School, Boston, Massachusetts, United States of America; 2 Socios En Salud Sucursal Perú, Lima, Peru; 3 Boston University School of Public Health, Department of Biostatistics, Boston, Massachusetts, United States of America; 4 Brigham and Women’s Hospital, Division of Global Health Equity, Boston, Massachusetts, United States of America; Baldwin Wallace University, UNITED STATES

## Abstract

**Objective:**

To use routinely collected data, with the addition of geographic information and census data, to identify local hot spots of rates of reported tuberculosis cases.

**Design:**

Residential locations of tuberculosis cases identified from eight public health facilities in Lima, Peru (2013–2018) were linked to census data to calculate neighborhood-level annual case rates. Heat maps of tuberculosis case rates by neighborhood were created. Local indicators of spatial autocorrelation, Moran’s I, were used to identify where in the study area spatial clusters and outliers of tuberculosis case rates were occurring. Age- and sex-stratified case rates were also assessed.

**Results:**

We identified reports of 1,295 TB cases across 74 neighborhoods during the five-year study period, for an average annual rate of 124.2 reported TB cases per 100,000 population. In evaluating case rates by individual neighborhood, we identified a median rate of reported cases of 123.6 and a range from 0 to 800 cases per 100,000 population. Individuals aged 15–44 years old and men had higher case rates than other age groups and women. Locations of both hot and cold spots overlapped across age- and gender-specific maps.

**Conclusions:**

There is significant geographic heterogeneity in rates of reported TB cases and evident hot and cold spots within the study area. Characterization of the spatial distribution of these rates and local hot spots may be one practical tool to inform the work of local coalitions to target TB interventions in their zones.

## Introduction

Tuberculosis (TB) remains one of the leading infectious killers in the world, with large populations facing high and stagnant case rates of this preventable disease [1]. An emerging network of coalitions—the Zero TB Initiative—seeks to rapidly drive down TB case rates in geographically defined zones, by locally deploying simultaneous strategies to increase: case finding, access to treatment for all forms of TB disease, and access to TB preventive treatment [2]. One key step toward closing gaps in case detection will be to characterize the baseline or starting point for not only the numbers of TB cases in a zone, but how they concentrate spatially so that the impact of interventions can be appropriately measured.

The geographic distribution of TB is variable at global-, country-, district-, and community-levels. Geographic heterogeneity of TB indicates an uneven distribution of disease burden across spatial scales [1, 3]. Characterization of the spatial distribution of TB cases and high-risk areas may serve as a practical tool for policy makers and can inform public health responses, such as targeting and tailoring interventions [4]. To design effective interventions for local zones, there is a need for finer spatial resolution of TB data—thus a shift from only analyzing nationally aggregated data to using the relevant local data. Essential to this shift is the improved use of existing data, such as program reports of TB cases, and the expansion of those routine data to include geographic information [4]. Previous studies have demonstrated how evaluation of spatial variations of TB disease can identify high-risk areas to be spatially targeted with screening interventions [5].

A systematic review of methods used in spatial analyses of TB reported that these typically have used aggregated case data, as opposed to individual-level case data (78% vs 22%), over administrative spatial units, and that the scale of spatial aggregation varied greatly [6]. For example, different analyses have used units at the continent-, country-, province-, district-, state-, county-, neighborhood-, census tract-, postal code-, health area-, municipality-, government area-, and ward-level [6]. Geographic variability in rates of TB was found regardless of the scale used. In order to guide local tailoring and targeting of interventions, however, it is essential to characterize TB case data on relevant smaller scales, as other topic areas have demonstrated [7]. As part of the design phase of a local coalition’s TB case finding initiative [8], we sought to use routinely collected data, adding geographic information and census data, to identify local hot spots of reported TB case rates.

## Materials and methods

### Study setting

**Fig 1:**
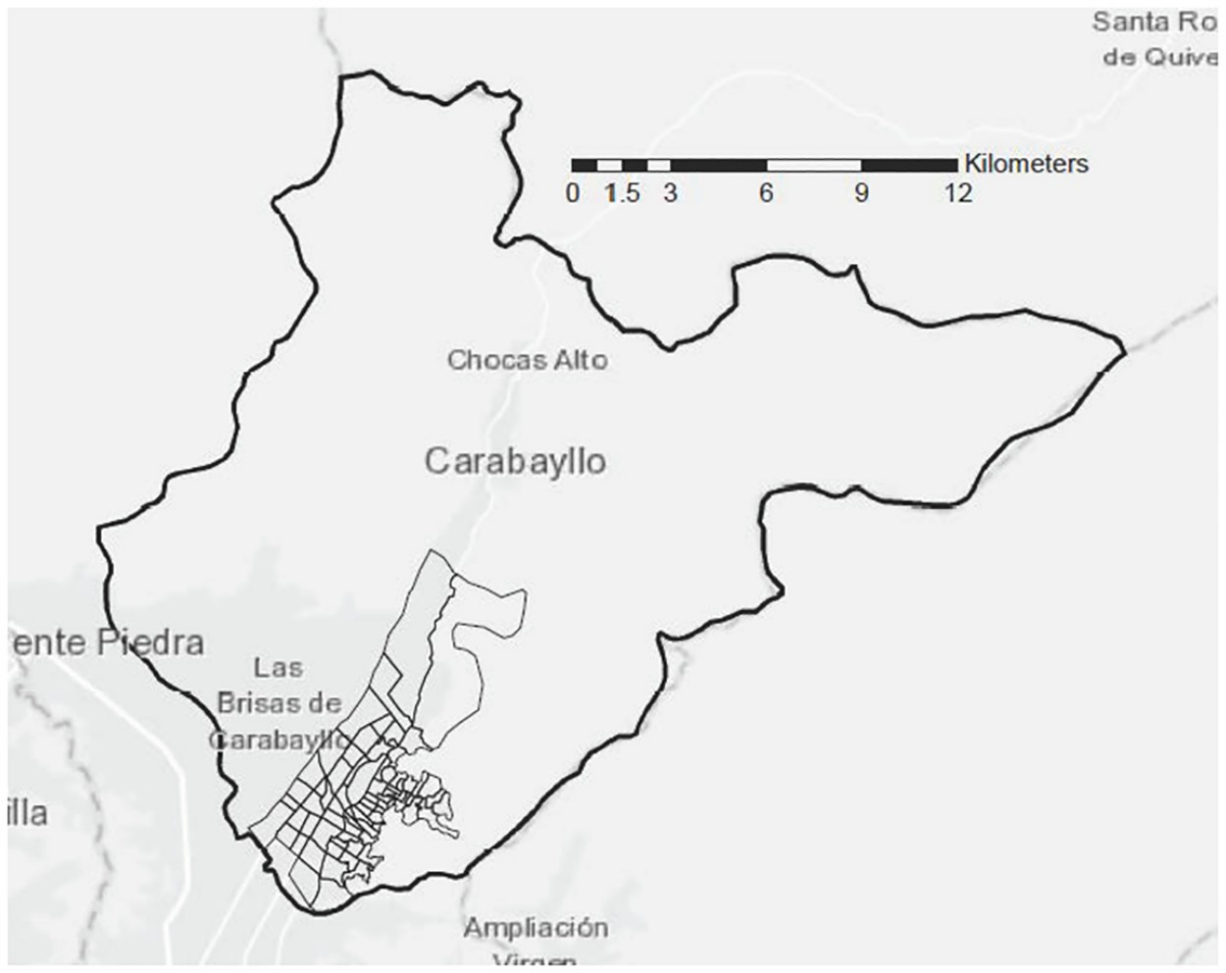
Study area within the Carabayllo District, Lima, Peru. The “Light gray canvas” basemap from ESRI (Environmental Systems Research Institute, Redlands, California, USA; https://www.esri.com/en-us/arcgis/products/arcgis-desktop/) was used to create this figure.

Peru is a middle-income country with a population of 32 million people and an estimated TB incidence of 119 (91–150) per 100,000 population in 2019 [9]. Our study took place in the contiguous catchment areas of eight primary-level public health facilities in Carabayllo district, the northernmost district of metropolitan Lima, Peru. This is an urban zone (Fig 1), which was home to approximately 209,000 inhabitants in 2017, comprising 64% of the district’s population [10]. The catchment areas of the four other health facilities in Carabayllo were excluded from this analysis because we could not readily access health center data about TB case reports nor accurate census data for that catchment population.

### Data collection and measures

We collected data about patients treated for TB disease between 2013 and 2017 from eight primary-level public health centers in a contiguous geographic region of Carabayllo. To access free TB treatment provided routinely by the Ministry of Health services, patients receive their TB treatment doses at the health facility in whose catchment area their residence is located. While Peru has a national electronic TB surveillance system, the electronic records of the health centers’ TB treatment registers in the study area were inaccessible. Therefore, we reviewed the paper TB treatment registers at the eight health centers and extracted age and sex recorded for each TB patient into an electronic data abstraction form. If age or sex were missing, the patient’s paper medical chart was reviewed. We then used Google Maps to determine an approximate geographic coordinate for each patient’s address listed in the treatment register and recorded the coordinate, in the form of latitude and longitude, in an electronic data form. We used this approach to assign geographic coordinates because many neighborhoods in this district do not use a standard address system. In consultation with community leaders, we defined boundaries that correspond to locally defined neighborhoods within the study area and created maps and then shapefiles of these neighborhoods.

Additionally, we obtained the 2017 census data for the Carabayllo district from the National Institutes of Statistics and Information in Peru [10]. The census data are aggregated by block-level tracts; within each tract, population numbers are available in aggregate and also stratified by age and sex. Because the block-level census tracts do not align completely with the local neighborhood boundaries, and the blocks are substantially smaller than the neighborhoods, we calculated the proportion (by area) of each block-level census tract that fell into each neighborhood. We then multiplied that proportion by the population provided for that census tract and summed across each census tract that overlapped with the neighborhood to obtain the total population in each neighborhood. We applied the same proportions to other census data—including population by sex and age group (less than 15 years, 15–44 years, and greater than 44 years)—to obtain population breakdowns by these characteristics across each neighborhood.

Finally, we overlaid the geocoded addresses of each TB case onto the neighborhood-level map. Any individual whose residence lay outside of the neighborhood-level map was excluded from this analysis. We then estimated the historic rates of reported TB cases from 2013–2017 for each neighborhood by dividing the total number of individuals with TB in each neighborhood by the 2017 estimated population calculated in each neighborhood. We then divided by five to give an average annual rate per 100,000 population for the five-year period to account for variability during this time frame. In addition to the rates of the total reported cases, we calculated age- and sex-specific rates by dividing the TB cases with each characteristic in each neighborhood by the 2017 estimated population for that age group or sex in each neighborhood. To visualize rates of reported TB cases, we created heat maps, which are graphical representations of the data where values are depicted by variations in hue.

### Analysis

We calculated the median, interquartile range (IQR), and range of the annual rates of reported TB cases over the 74 neighborhoods, in aggregate and by age and sex for each neighborhood. We produced heat maps to visually display the geographic variability of rates over the study area.

The global univariable Moran’s *I* was used to test for the presence of spatial clustering (spatial autocorrelation) in rates in the entire study area; we calculated the Moran’s *I* and pseudo p-value based on 999 permutations. The null hypothesis assumes complete spatial randomness. We calculated Local Indicators of Spatial Autocorrelation (LISA) [11] to identify and locate clusters of neighborhoods with a relatively high or low rate. A first order queen contiguity connectivity matrix was used to define neighbors. For each neighborhood, we calculated a LISA statistic—a localized Moran’s *I*—to give indication of the extent of the presence or absence of spatial dependency. For each neighborhood, we conducted hypothesis tests using the Moran’s *I*, where the null hypothesis assumed that rates in each neighborhood were spatially independent from rates in surrounding contiguous neighborhood units. All calculated localized Moran’s *I*s controlled for a false discovery rate, which is a known method to account for multiple and dependent tests in calculating LISA statistics.

Cluster maps were produced to visualize the neighborhoods with a significant local LISA statistic at the p≤0.05 level and to indicate the type of spatial associations observed. Each neighborhood with a significant LISA statistic was then classified as either a spatial cluster or a spatial outlier in relation to the surrounding neighborhoods. Spatial clusters include hot spots and cold spots, which are defined as a neighborhood with a higher or lower, respectively, concentration of events compared to the expected number given a random distribution of events. Spatial outliers are neighborhoods that have rates that are markedly different from that of their spatial neighbors; a high spatial outlier is a neighborhood with a high rate surrounded by neighborhoods of low rates, whereas a low spatial outlier is a neighborhood with a low rate surrounded by neighborhoods of high rates. The cluster maps visually display the neighborhoods according to these spatial clustering or spatial outlier classifications. Maps were created for the overall neighborhood rates, as well as age- and sex-stratified rates.

MBB created all maps using ArcMap Desktop version 10.8 (Environmental Systems Research Institute, Redlands, California, USA; https://www.esri.com/en-us/arcgis/products/arcgis-desktop/).

### Ethics statement

Data collected in Peru were part of programmatic clinical documentation at health centers for patients diagnosed with tuberculosis, thus local Institutional Review Board approval was not required for the collection of this data, nor did patient provide written informed consent for participation. Patient records were accessed between December 2017 and March 2018. Patients whose records were accessed sought treatment for TB between January 2013 and December 2017. All patient data were fully anonymized before they were accessed and used for analysis. This study was determined to constitute exempt human subjects research by the Mass General Brigham Institutional Review Board because it involved only secondary use of programmatic data in a manner that did not allow identification of individual participants.

## Results

Between 2013 and 2017, a total of 1,323 individuals received treatment for TB disease at eight health facilities in the study area, which comprises 74 contiguous neighborhoods. The total study area was 27.8 square kilometers, with a median area in square kilometers for the individual neighborhoods of 0.21 (IQR: 0.13–0.35, range: 0.04–4.36). We excluded 28 (2.1%) of these individuals because their residential addresses were located outside the study area. Of the 1,295 individuals included, 814 (62.9%) were male, 96 (7.4%) were less than 15 years old, 921 (71.1%) were 15–44 years old, and 278 (21.5%) were greater than 44 years old.

The total rate of reported TB cases in the study area was 124.2 per 100,000 population; notably, this aggregated statistic masks substantial geographic heterogeneity. When assessing rates by neighborhood, the median annual rate was 123.6 per 100,000 population. This varied greatly by neighborhood, ranging from 0.0 to 800.0 per 100,000 population (Table 1). We observed similar geographic heterogeneity for age- and sex-specific rates.

**Table 1:** Variability in annual rates of reported TB cases (per 100,000 population) among 74 neighborhoods.

	Median	Interquartile Range	Range
**Total population**	123.6	58.9–185.6	0–800.0
**Age <15 years**	23.4	0–42.7	0–148.7
**Age 15–44 years**	159.4	85.5–267.0	0–766.3
**Age >44 years**	80.5	34.9–162.6	0–2222.2
**Men**	157.3	74.7–238.1	0–800.0
**Women**	83.3	42.8–138.2	0–800.0

**Fig 2:**
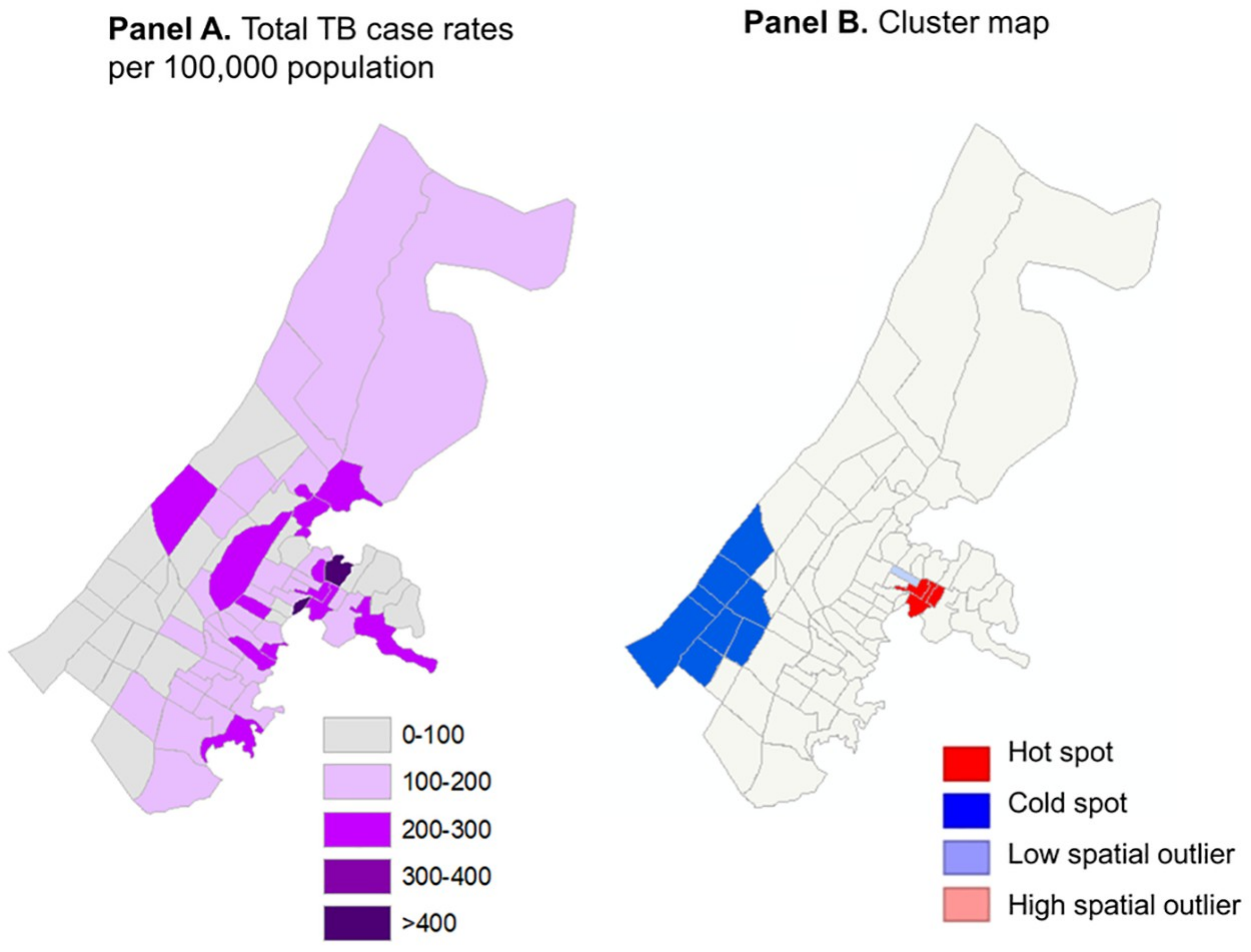
Geographical variation and spatial clustering of rates of reported TB cases (per 100,000 population).

Moderate spatial clustering of annual rates was observed in the study area, as indicated by a Moran’s *I* of 0.144, *p* = 0.021. In the south-western region of the study area, clusters of low rates (cold spots) were observed, whereas in the eastern-central region a cluster of high rates (hot spots) were identified (Fig 2). A single low spatial outlier (neighborhoods with low rates surrounded by those with high rates) was also identified in the eastern-central region, and no high spatial outliers were observed.

**Fig 3:**
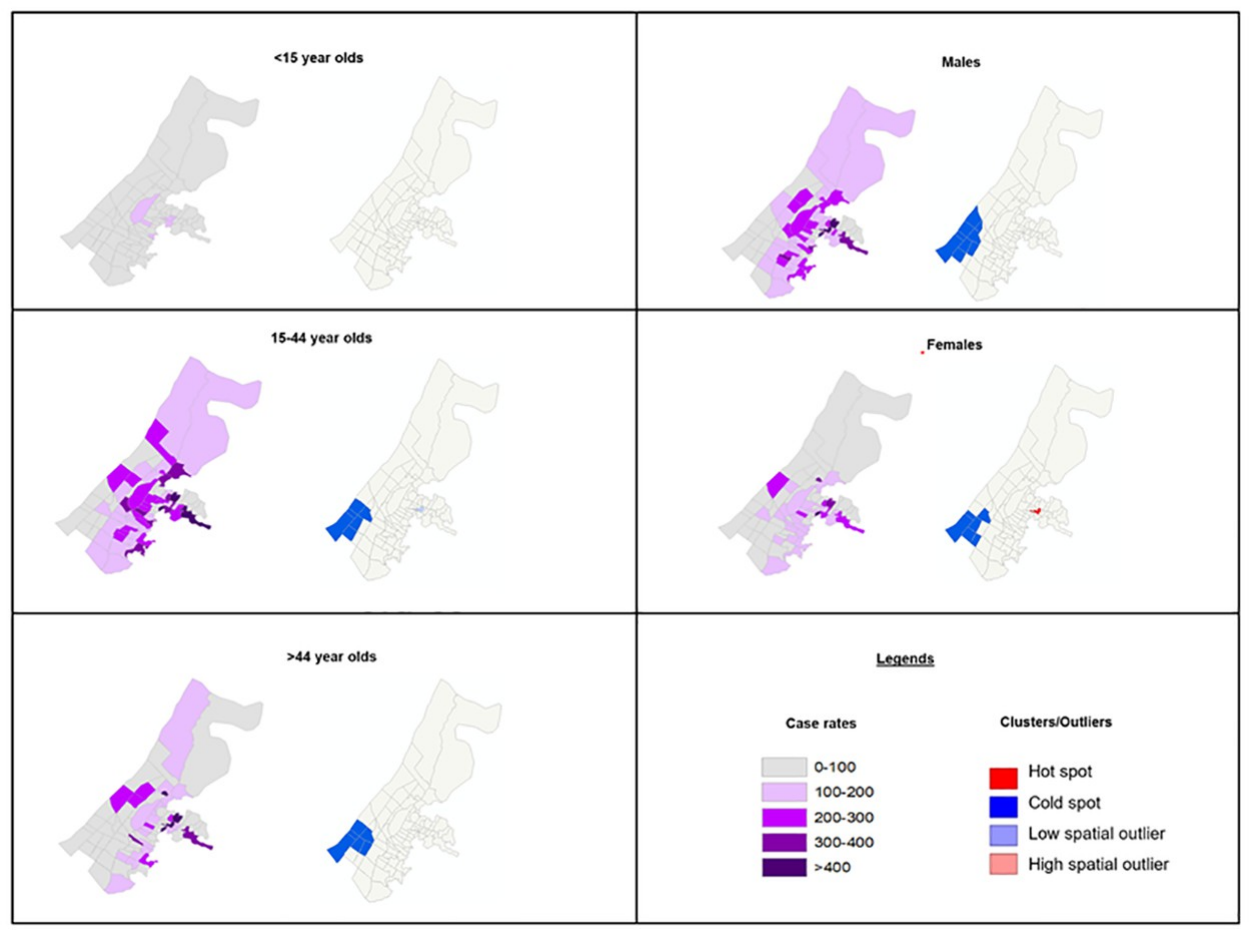
Geographical variation and spatial clustering of age- and sex-specific rates (per 100,000 population).

We observed no spatial clustering of rates stratified by age group (age <15: Moran’s *I* = -0.047, *p* = 0.337; age 15–44: Moran’s *I* = 0.079, *p* = 0.084; age >44: Moran’s *I* = 0.058, *p* = 0.070). However, we did find moderate spatial clustering of sex-specific annual rates (men: Moran’s *I* = 0.138, *p* = 0.031; women: Moran’s *I* = 0.110, *p* = 0.036). For the 15–44 and greater than 44 age groups and both sexes, we observed a cold spot in the south-western region of the study area; the cold spot is larger for males than females (Fig 3). We also observed a hot spot in the eastern-central region of the study area for females.

## Discussion

We found significant heterogeneity in the rates of reported TB cases within this district of northern Lima, Peru. Characterization of reported TB case rates over smaller spatial scales allowed for identification of geographic areas with more and less concentrated TB risk; these hot spots and cold spots were not apparent from the case notification rates measured at a district scale. Overall, the total rate for the study area (comprising around two thirds of the district) was 124 per 100,000 population, which is only slightly higher than the estimated national TB incidence of 119 per 100,000 population in 2019 [9]. However, when we looked at the neighborhood-level rates, we observed great variability across neighborhoods with rates ranging from 0 to 800 per 100,000 population. This points to the absence of a uniform risk of TB within this focal urban zone of 209,000 inhabitants. It also highlights that looking at rates of reported TB cases over smaller geographic units can uncover high-risk and low-risk zones that are not apparent even at the district level.

By using routinely collected data—TB registers and the census—and adding the geographic coordinates to each reported case, we were able to calculate rates of reported TB cases and then create simple maps to identify hot and cold spots within a geographic zone of interest. Insights from the neighborhood-level heterogeneity of reported case rates can have great implications for program planning. While some interventions are known to find TB cases, such as household contact investigations which identify individuals exposed at home to TB [1], those interventions will not find the majority of those sick with TB, since less than twenty percent of TB transmission is thought to take place within households [12, 13]. Thus, geographic analyses on routinely collected data can be used to identify zones in a community where TB cases or risk factors for transmission are concentrated, in order to help public health workers discern where community-based screening efforts may be deployed [3, 4, 14]. Additionally, areas identified as cold spots may point to zones where there may be a lack of diagnostic services.

In this study, we observed relatively higher rates of reported TB cases among residents in the age group of 15–44 years old and among males. This finding is consistent with previous observations in Peru, in which TB diagnoses were disproportionately higher among those 15–44 years old (made up 66% of case notifications) and among males (61% of case notifications) [15]. Conducting age- and sex-stratified analyses may identify important differences in other settings. If different hot spots for these subgroups are discerned—as observed by a hot-spot for women and not men—this would allow for tailored approaches to detect more cases in these subgroups, especially since working-age adults and men may face distinct challenges for seeking or accessing TB care [16].

Admittedly, there are many challenges in interpreting spatial clustering of high rates of reported TB cases. Most notable is that we are unable to decipher whether the observed geographic heterogeneity in rates of reported TB cases is driven by true spatial variation of TB prevalence, by care-seeking behaviors of the underlying population, or by access barriers to diagnostic and treatment facilities [17, 18]. Spatial variation in reported TB rates may also be influenced by the quality of the data collected and the type of spatial analysis method used [6]. For example, our study relied on data in the paper TB treatment registries at local facilities during a period where passive case-finding was the norm; these registries would thus be expected to miss a large number of TB cases and may skew the observed clusters towards areas with better access to diagnostic services [1, 18]. Additionally, we cannot conclude that observed hot spots reflect TB transmission; this is because we used residential location to identify in which neighborhood the TB cases resided, but it is possible that TB transmission occurred in other zones where patients work, commute, or gather socially. These limitations notwithstanding, our approach can serve to identify local zones that should be considered for targeting case-finding interventions.

There are several other limitations to note. We used the 2017 census data to calculate the populations of each neighborhood. In order to compute more accurate rates, it would have been ideal to calculate projected populations for each year that corresponded to the collected TB case data (2013–2018). That approach to population estimation is standard practice for most National Statistics Bureaus, namely to estimate populations for each of the years between censuses. However, we were unable to use this approach because the study district has geographically expanded in the past decade due to migration, leading to the census tracts being completely redrawn from the time of the previous census in 2007. Thus, we are unable to distinguish whether shifts in rates of reported TB cases over time are due to changes in true TB risk, TB case reporting, or the expanding population across the district.

Finally, it is important to reiterate that the case rates analyzed here may not represent the true TB disease burden in this community or its geographic distribution. There are several factors that lead to a likely underestimation of that true underlying disease burden. First, simply using the rates of cases identified through a treatment register is known to underestimate the true burden of TB cases in a zone, given that during the study period, the health centers relied on sputum smear microscopy, which has a low sensitivity [19], as well as on passive case finding. Thus, the reported cases do not include those individuals who did not seek care or those who did seek care but were misdiagnosed. A second reason that the rates we analyzed likely underestimate the true burden of disease is that we only used case reports at the Ministry of Health’s health centers in the district. These reports do not account for patients treated by a second network of government hospitals that covers district residents with employer-based insurance and which has recently expanded its TB services. Additionally, the counts of reported cases that we used did not include individuals who live in the study district but received TB treatment at referral hospitals outside the district.

Geospatial techniques can be applied to identify spatial variability of reported TB case rates, which can in turn provide critical information for program planning. It is unknown whether the observed heterogeneity in reported TB case rates is due to underlying differences in disease burden or in barriers to accessing TB diagnostic services. But comparison of neighborhood-level rates of reported TB cases is important to identify neighborhoods and zones that merit closer evaluation as potential targets for intensified case-finding efforts. Local coalitions working to close gaps in TB case detection can use routinely collected data, adding geospatial information and census data, as a first step to inform new collective actions.
